# Coverage and inequalities in maternal and child health interventions in Afghanistan

**DOI:** 10.1186/s12889-016-3406-1

**Published:** 2016-09-12

**Authors:** Nadia Akseer, Zaid Bhatti, Arjumand Rizvi, Ahmad S. Salehi, Taufiq Mashal, Zulfiqar A. Bhutta

**Affiliations:** 1 Centre for Global Child Health, Hospital for Sick Children, Toronto, Canada; 2 Dalla Lana School of Public Health, University of Toronto, Toronto, Canada; 3 Center of Excellence in Women and Child Health, the Aga Khan University, Karachi, Pakistan; 4 London School of Hygiene & Tropical Medicine, London, UK; 5 Afghanistan Ministry of Labor, Social Affairs, Martyrs and Disabled, Kabul, Afghanistan; 6 Former Ministry of Public Health, Kabul, Afghanistan

**Keywords:** Afghanistan, Maternal, Child, Interventions, Health, Equity, Equality

## Abstract

**Background:**

Afghanistan has made considerable gains in improving maternal and child health and survival since 2001. However, socioeconomic and regional inequities may pose a threat to reaching universal coverage of health interventions and further health progress. We explored coverage and socioeconomic inequalities in key life-saving reproductive, maternal, newborn and child health (RMNCH) interventions at the national level and by region in Afghanistan. We also assessed gains in child survival through scaling up effective community-based interventions across wealth groups.

**Methods:**

Using data from the Afghanistan Multiple Indicator Cluster Survey (MICS) 2010/11, we explored 11 interventions that spanned all stages of the continuum of care, including indicators of composite coverage. Asset-based wealth quintiles were constructed using standardised methods, and absolute inequalities were explored using wealth quintile (Q) gaps (Q5-Q1) and the slope index of inequality (SII), while relative inequalities were assessed with ratios (Q5/Q1) and the concentration index (CIX). The lives saved tool (LiST) modeling used to estimate neonatal and post-neonatal deaths averted from scaling up essential community-based interventions by 90 % coverage by 2025. Analyses considered the survey design characteristics and were conducted via STATA version 12.0 and SAS version 9.4.

**Results:**

Our results underscore significant pro-rich socioeconomic absolute and relative inequalities, and mass population deprivation across most all RMNCH interventions studied. The most inequitable are antenatal care with a skilled attendant (ANCS), skilled birth attendance (SBA), and 4 or more antenatal care visits (ANC4) where the richest have between 3.0 and 5.6 times higher coverage relative to the poor, and Q5-Q1 gaps range from 32 % - 65 %. Treatment of sick children and breastfeeding interventions are the most equitably distributed. Across regions, inequalities were highest in the more urbanised East, West and Central regions of the country, while they were lowest in the South and Southeast. About 7700 newborns and 26,000 post-neonates could be saved by scaling up coverage of community outreach interventions to 90 %, with the most gains in the poorest quintiles.

**Conclusions:**

Afghanistan is a pervasively poor and conflict-prone nation that has only recently experienced a decade of relative stability. Though donor investments during this period have been plentiful and have contributed to rebuilding of health infrastructure in the country, glaring inequities remain. A resolution to scaling up health coverage in insecure and isolated regions, and improving accessibility for the poorest and marginalised populations, should be at the forefront of national policy and programming efforts.

**Electronic supplementary material:**

The online version of this article (doi:10.1186/s12889-016-3406-1) contains supplementary material, which is available to authorized users.

## Background

The recent Millennium Development Goals (MDG) period experienced considerable advances towards achieving social and economic equity. One such effort, championed by the Countdown to 2015 (Countdown) consortium [1], steered the compilation and broad dissemination of current information on country progress in maternal, newborn and child health and survival, essential interventions coverage, and equality. Countdown focused on 75 of the highest burden countries globally, and also commissioned provocative in-depth country case studies on fragile and vulnerable nations, including Afghanistan.

Afghanistan is an exceedingly poor nation of about 30 million located in South-Central Asia. With a human development index (HDI) of 0.47 in 2014, ranking it 171 among 188 nations, the country has some of the worst health indicators worldwide [2]. Plagued by over three decades of widespread war and unstable governance since 1979, Afghanistan continues to be in a delicate and volatile state today.

Results from Countdown’s country case study revealed important improvements in Afghanistan since the collapse of the Taliban regime in 2001. Best modeled estimates highlight dramatic gains in MDG5 with maternal mortality rates (MMR) dropping from 1100 to 396 deaths per 100,000 live births from 2000 to 2015 [3]. Over the past 15 years, MDG4 progress has also been notable with under 5 child mortality rates (U5MR) reducing 34 % (from 137 to 91 deaths per 1000 live births) and newborn mortality rates (NMR) dropping 32 % (from 53 to 36 deaths per 1000 live births) [4]. In line with survival gains, the country has also managed to increase coverage of indispensable maternal and child public health interventions to its populations [5].

Despite promising efforts at the national level, accelerated improvement is restricted by huge subnational disparities. Inequities exist in access to and utilisation of many preventative and curative health services, and are particularly exacerbated between the richest and poorest, and across geographical regions in Afghanistan [6–9]. A key element of the Sustainable Development Goals (SDGs) is to effectively mitigate inequalities and ensure universal coverage of essential preventative interventions [10]. Goal 10 is specifically intended to “reduce inequality within and among countries”. Ambitious targets have been set to achieve and sustain income growth among the poorest populations, and to empower and promote social, economic, and political equity in national strategies. In line with these goals, Afghanistan will need to better understand inequalities in order to prioritise effective and equitable strategies for improving women’s and children’s health. This is also a key mandate of the Ministry of Public Health’s (MoPH) strategic direction for Afghanistan [11]. Efforts focused on reducing inequities will prove valuable for sustaining and scaling up gains in Afghanistan in the post-2015 era.

While inequalities are ubiquitous and a reality of life, the concept of inequity refers to the degree of unfairness and injustice in societies which often result from pervasive inequalities. In this study we hold that societal inequities may result from unjust inequalities in services/products available to and accessed by populations. We uphold this distinction between equality and equity for the current study. Socioeconomic and regional inequalities in Afghanistan have been recognised in the literature, specifically in relation to maternal and child health [12–14], mortality [13, 15, 16], interventions coverage [13], and health service utilisation [7–9, 17, 18]. However, available reports largely assessed socioeconomic position using single proxy measures such as income or education. Others have developed the composite asset index which has been shown as more indicative of actual wealth in low and middle income countries (LMIC) [19]. Among those that employed the asset index, inequalities between wealth groups were largely examined in the form of gaps and ratios, without consideration of robust measures that take into account the cumulative population wealth distribution. Yet, further available assessments have not distinguished between absolute versus relative socioeconomic inequalities. Lastly, in Afghanistan, no studies have examined wealth inequality across a whole range of essential reproductive, maternal, newborn and child health (RMNCH) interventions nor spatially examined these at region-level specificity.

Using recent national data from Afghanistan, this study aims to explore the following objectives: a) assessing levels of coverage, and the absolute and relative socioeconomic inequalities in 11 essential RMNCH interventions, including measures of composite coverage, at the national level and for the eight geographic regions of the country; b) quantifying the number of child deaths averted through scale up of effective community-based interventions across socioeconomic groups using the Lives Saved Tool (LiST) [20].

## Methods

### Data source

**Fig. 1:**
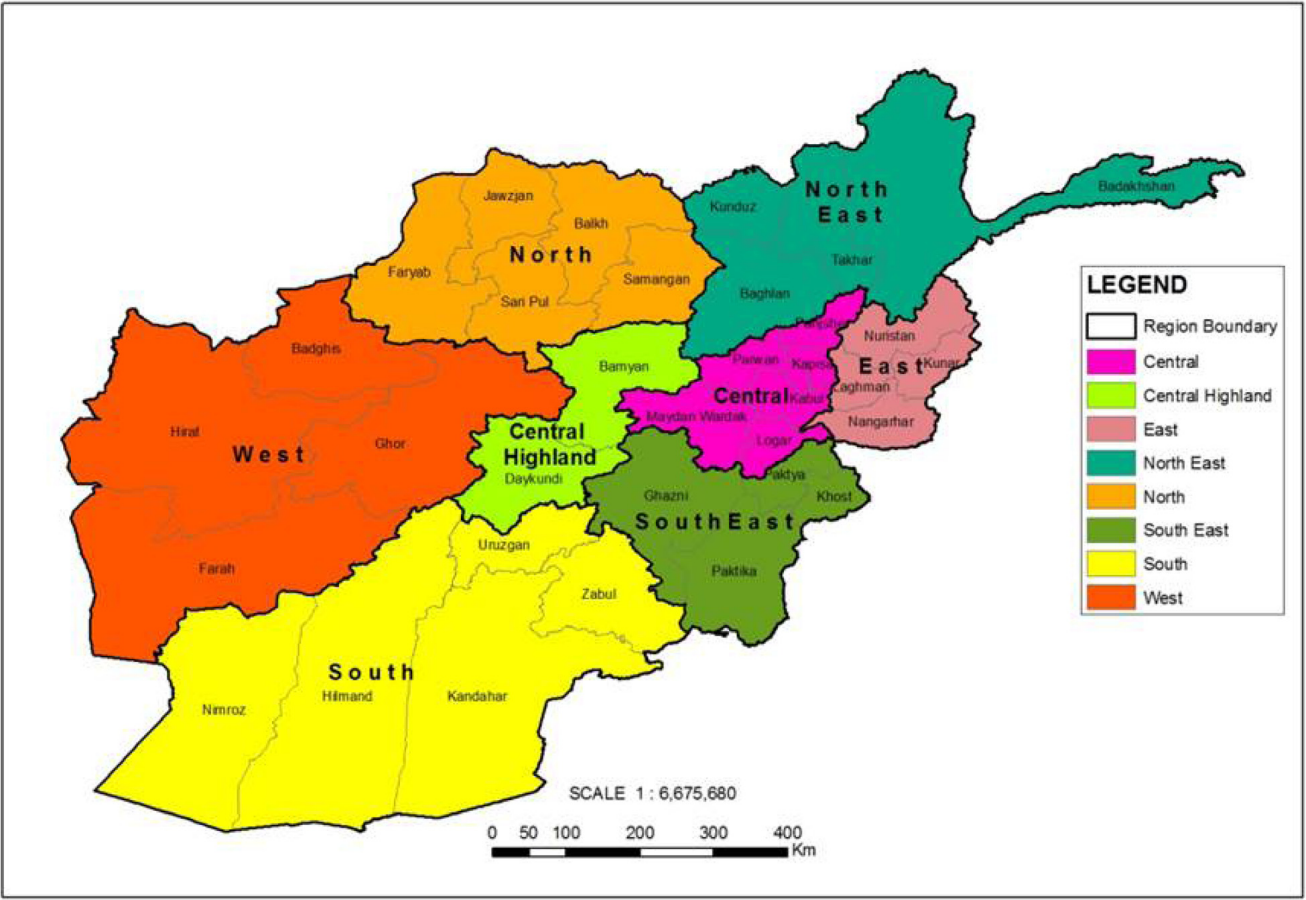
Geographical spread of 8 regions and 34 provinces of Afghanistan. Note: Regions are color coded as presented in the legend. Provinces are clustered within the regional zone

We used data from the 2010/2011 Multiple Indicator Cluster Survey (MICS), a recent large-scale health and nutrition survey conducted in Afghanistan [21]. The survey is both nationally and regionally representative, contains sufficient data on a range of interventions across the continuum of care, and also includes information on household assets to compute the wealth indices. Data was obtained through interviews with household members. We analyzed data from 21,290 women of reproductive age (15–49 years) and a total of 14,872 children between the ages of 0–59 months. We studied trends in the eight geographic regions of Afghanistan (Fig. 1), namely Central, Central Highlands, East, North, North East, South, South East and West. We calculated regional estimates rather than by the 34 provinces to ensure adequate power and precision of estimates.

### Selection of interventions

We selected a range of preventative and curative interventions that spanned all stages of the continuum of care. The indicators varied in high and low coverage in Afghanistan and were diverse in delivery mechanisms (e.g. administered via health services, the community delivery platforms and mass education campaigns). Interventions included the following: family planning needs satisfied (FPS), antenatal care with a skilled provider (ANCS), 4 or more antenatal care visits (ANC4), skilled attendant at birth (SBA), early initiation of breastfeeding [within one hour] (EIBF), 3 doses of DPT vaccine (DPT3), measles vaccination (MSL), full immunisation of children (FULL), vitamin A supplementation (VITA), oral rehydration therapy and continued feeding for children with diarrhoea (ORT) and care seeking for children with suspected pneumonia (CPNM). Data for FPS was not available in the MICS 2010/2011 dataset so we used Boerma et al’s recommended equation to estimate family planning needs satisfied from contraceptive prevalence coverage [22]. All indicators were estimated according to standard Countdown definitions [1] and have been detailed in Additional file 1-A.

### Composite and co-coverage

We explored two summary measures of coverage- the co-coverage indicator [23] and the composite coverage index (CCI) [22]. Such composite indices are useful for comparative within-country, cross-country and time series analyses [22, 23]. The co-coverage indicator assesses a range of preventative public health interventions that have been proven to accelerate maternal, newborn and child survival. These include eight essential interventions: ANCS, tetanus toxoid 2+ doses during pregnancy (TT2), SBA, VITA, BCG, DPT3, MSL, and, household access to improved drinking water sources (WA). The co‐coverage is calculated as the number of interventions each mother/child pair received and therefore ranges from 0 to 8. We estimated the proportion of maternal and child pairs receiving any integer of interventions and plotted them by wealth quintiles to explore socioeconomic differentials. We also calculated the proportion of mothers/children receiving 3+ or 6+ essential interventions. TT2 and WA (detailed definition in Additional file 1-A) were included as part of co-coverage but were not evaluated separately.

Developed by Boerma et al [22], the CCI has been proposed as an alternate aggregate measure which includes both curative and preventative child and adult interventions. The CCI was also intended for multi-context and over time studies, and has been used widely [22, 24, 25]. The composite measure is calculated as a weighted coverage mean of eight essential interventions that represent broad categories of the continuum of care. The four categories are as follows: family planning, maternal and newborn care, immunization, and case management of sick children. Each continuum stage is given equal weight and the CCI is then calculated as:

$$ CCI=\raisebox{1ex}{$1$}\!\left/ \!\raisebox{-1ex}{$4$}\right.\left(FPS+\frac{SBA+ ANCS}{2}+\frac{2DPT3+MSL+BCG}{4}+\frac{ORT+ CPNM}{2}\right) $$

In addition to indicators that were previously defined, the CCI analysis uses coverage of BCG vaccination among children (BCG) (defined in Additional file 1-A).

The co-coverage and CCI were consulted to get a sense of the nation’s overall performance in access and delivery of life-saving interventions to the child and mother. Additionally, we use these aggregate measures to compare and rank interventions coverage across geographic regions.

### Equity analyses

We examined socioeconomic position using the asset index, a measure that has clear advantages over indicators such as income and education [19] and is the preferred way of measuring wealth in LMICs [26]. Using household asset data, we tabulated wealth index scores using principal components analysis and standardised methods [27]. Household scores were assembled into wealth quintiles where the lowest quintile (Q1) represented the poorest 20 % of the population and the highest quintile (Q5) represented the richest fifth. Inequalities may be expressed in both absolute and relative terms. Relative indicators provide insight into the degree of unfairness between the richest and poorest, while absolute measures provide an idea of the actual gap that exists between the groups and thus the effort required to close it. Exploring these in tandem is essential to revealing the full picture of inequality [28–30], and we’ve explored both types in this study. We considered at one simplistic and one sophisticated measure to explore each of absolute and relative socioeconomic inequalities. Simple indices included the absolute gap (calculated as the coverage gap between the poorest and richest quintiles [Q5-Q1]) and the relative ratio (estimated as the ratio of coverage in the richest to the poorest quintiles [Q5/Q1]). Advanced measures of socioeconomic inequality included the absolute slope index of inequality (SII) and the relative concentration index (CIX). The merits of the SII and CIX over coverage gaps and ratios, respectively, are well documented [30, 31]. The SII and CIX indices are weighted for population size and take into account any changes in the ordinal categories of the socioeconomic marker (e.g. differentials between wealth quintiles 2, 3 and 4), while the simplistic gap/ratio computations disregard sample sizes and intermittent values. Gaps and ratios however have a more intuitive interpretation and are easy to convey to lay audience and public health experts [19]. Due to the advantages and limitations set by the different measures, it has been recommended that both types of indicators be estimated when communicating to broad audiences [19].

The SII and CIX were calculated with appropriate standard errors and corresponding 95 % confidence intervals using standardised methods [19, 31]. As an example, the computation of SII ensued via logistic regression (to constrain percentages between 0–1) where the RMNCH indicator of interest was regressed onto the midpoint values of wealth quintiles that cover the cumulative population distribution. The difference in predicted values of the highest and lowest quintile (Q5 - Q1) generated the SII, interpreted as the percentage point difference between the richest and the poorest. Considering this SII formulation, positive values correspond to the intervention being higher in the wealthier subgroup, whereas negative values imply higher coverage in the poorest subgroup; a value of 0 indicated absence of absolute inequality using this metric. The relative inequality index CIX was calculated using analogous methods [19]. The theoretical maximum for CIX is ±1, where 0 indicates no relatively inequality and values closer to +1 favour the rich (while values closer to −1 favour the poor); we multiplied CIX values by 100 for presentation. For our study, we grouped CIX (values) and SII (%) into low (<|15|), moderate (|15-40|), high (|40–60|), and very high (>|60|) categories of socioeconomic inequality.

### Regional analyses

To investigate regional inequalities, we examined the CIX and SII measures for SBA, measles, and the co-coverage (6+ interventions) indicators. We focused primarily on inequality measures that take into account the whole population such as CIX and SII as it has been recommended for comparisons across geographic regions [19]. SBA and measles interventions were selected as they depend on different delivery channels, which may have implications to equitable access and reach; birth with a skilled attendant requires an accessible and functioning health system, and measles vaccination is often delivered through mass immunization campaigns, sometimes door to door and requires only one dose. We also assessed the co-coverage of 6+ interventions as it conveys an overall measure of access to 8 essential interventions and permits analysis of individual level data which could be exploited by region [23]. The co-coverage assesses only preventative interventions and though including curative services would have been desirable, this was not possible since curative interventions rely on two-week recall data from children who were ill just before the survey, and the resulting sample sizes would have been too small for equity analysis by region.

### Statistical analysis

Methods used to estimate lives saved using the Lives Saved Tool are presented in Table 1. We took into account the sampling design characteristics of the MICS survey and used STATA version 12.0 and SAS version 9.4 for all analyses. Final estimates of inequality and confidence bands were summarised in tabular and graphical formats as required. ArcView GIS software was used to visualise spatial patterns across provinces and regions. Ethics procedures were the responsibility of the institutions that commissioned, funded, or administered the survey.

**Table 1:** Lives Saved Tool (LiST) Methods

The Lives Saved Tool is a modelling software which has been used extensively over the past 10 years to estimate the potential impact of scaling up community and facility based interventions on mortality [20]. We used this analysis to assess the impact of community based interventions on neonatal and post neonatal mortality at the wealth quintile level. Data from the MICS 2010 were used for baseline estimates of health status, mortality and intervention coverages as required for LiST modeling. Current cause of death structure by wealth quintiles were not available for Afghanistan and we thus used LiST to compute these following procedures as per the method of Amouzou et al [32]. We modelled the impact of interventions amenable to scale up through first level health services and community platforms to reach deprived population sub-groups, as described in the recent childhood diarrhea and pneumonia [33] and nutrition series [34] for addressing inequities. A set of 12 interventions were scaled up from their most recent coverage level to 90 % by the year 2025 employing community-based approaches targeting the poor, usually rural and remote populations. Modelled interventions are listed in the box below.	
Impact on Neonatal or Post-neonatal Deaths	Effective Community-based Interventions
Both	Maternal micronutrient supplementation (iron, multiple micronutrients)
Both	Breastfeeding promotion
Post-neonatal	Complementary feeding promotion
Post-neonatal	Vitamin A supplementation
Post-neonatal	Promotion of hand washing practices
Neonatal	Chlorohexidine
Neonatal	Thermal care
Both	Oral rehydration solutions (ORS)
Post-neonatal	Zinc for diarrhea treatment
Neonatal	Oral antibiotics for treatment of neonatal infections
Post-neonatal	Oral antibiotics for treatment of pneumonia
Post-neonatal	Severe Acute Malnutrition (SAM) management -therapeutic feeding for severe wasting -treatment for moderate acute malnutrition

## Results

### Coverage and absolute/relative inequality

**Fig. 2:**
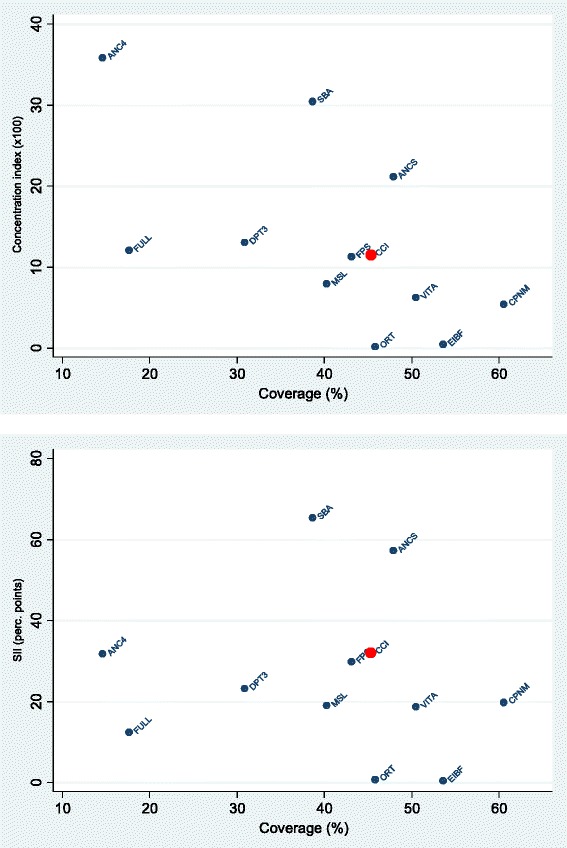
Comparison of relative (concentration index, *top*) and absolute (slope index of inequality, *bottom*) inequality between 11 preventive interventions plus the composite coverage index

Table 2 presents estimates of mean national coverage and absolute and relative inequality indices for 11 essential interventions and the CCI. Figure 2 visualises the coverage of interventions against SII and CIX inequality measures. The following results pertain to Table 2 and Fig. 2. Coverage levels of the majority of interventions was between 30–60 % with those ranking lower including DPT3 (31 %) and SBA (39 %). The highest coverage overall was noted for CPNM (61 %) while ANC4 (15 %) and full immunisation (18 %) were the least accessed interventions.

**Table 2:** Coverage and inequalities for essential interventions in Afghanistan

	Overall coverage (%)			Q1 coverage (%)			Q5 coverage (%)			Difference (Q5–Q1; % points)*			Slope index of inequality (% points)*			Ratio (Q5:Q1)*			Concentration index (×100)*		
	%	LCL	UCL	%	LCL	UCL	%	LCL	UCL	%	LCL	UCL	%	LCL	UCL	value	LCL	UCL	%	LCL	UCL
Family planning needs satisfied	43.1	42.3	43.9	35.8	34.0	37.6	60.7	59.1	62.3	24.9	24.7	25.1	29.8	13.7	46.0	1.70	1.66	1.74	11.3	5.0	17.6
Antenatal care with a skilled provider	47.9	44.6	51.1	25.8	21.2	30.4	78.1	74.6	81.5	52.2	51.1	53.3	57.2	50.5	64.0	3.02	2.68	3.51	21.1	18.2	24.1
Antenatal care visits (4+ visits)	14.6	12.7	16.5	5.8	3.4	8.2	32.4	28.6	36.1	26.5	27.9	25.1	31.7	26.2	37.2	5.57	4.41	8.31	35.9	30.0	41.8
Skilled birth attendant	38.7	35.3	42.0	15.6	12.0	19.1	76.3	72.8	79.9	60.8	60.8	60.8	65.4	60.0	70.8	4.91	4.18	6.06	30.5	27.3	33.7
Early start of breastfeeding	53.6	50.3	56.9	52.1	45.7	58.4	54.3	50.0	58.6	2.2	0.2	4.2	0.4	−8.3	9.1	1.04	1.00	1.09	0.5	−2.3	3.2
DPT3 immunisation	30.9	27.2	34.5	22.2	15.6	28.8	45.9	40.7	51.2	23.7	22.3	25.1	23.2	12.4	34.0	2.07	1.77	2.61	13.0	6.7	19.4
Measles immunisation	40.3	36.5	44.0	32.6	24.9	40.3	50.4	45.2	55.5	17.8	15.2	20.4	19.1	7.1	31.0	1.55	1.38	1.82	7.9	2.8	13.0
Full immunisation	17.6	14.4	20.9	13.2	7.0	19.4	23.9	19.4	28.5	10.8	9.1	12.4	12.4	2.0	22.8	1.82	1.47	2.78	12.1	1.5	22.6
Vitamin A in past 6 months	50.5	47.3	53.6	43.7	38.3	49.1	62.1	58.1	66.1	18.4	17.1	19.8	18.7	10.6	26.9	1.42	1.35	1.52	6.2	3.4	9.1
Oral rehydration therapy	45.8	41.2	50.4	47.0	39.7	54.3	40.8	35.4	46.2	−6.2	−8.1	−4.2	0.8	−8.6	10.1	0.87	0.85	0.89	0.2	−3.3	3.6
Care seeking for pneumonia	60.5	57.2	63.9	46.4	39.3	53.4	65.7	60.0	71.5	19.4	18.1	20.7	19.8	9.7	29.9	1.42	1.34	1.53	5.4	2.5	8.3
Composite coverage index	45.3	43.3	47.3	34.0	29.3	38.7	61.6	57.5	65.7	27.6	27.0	28.2	32.1	24.1	40.0	1.81	1.70	1.96	11.5	6.5	16.6

*Estimates presented as means with 95 confidence intervals

Note: The absolute difference in coverage levels of intervention between the richest (Q5) and poorest (Q1) populations is presented as the “Difference”; the “Ratio” is the measure of relative inequality that is calculated from coverage levels of Q5 divided by Q1. The Slope Index of Inequality is a measure of absolute inequality that includes the cumulative population distribution, and is interpreted as the percentage point difference between Q5 and Q1 (positive values correspond to the intervention being higher in the wealthier subgroup whereas negative values imply higher coverage in the poorest subgroup and a value of 0 indicated absence of absolute inequality). The Concentration Index is a measure relative inequality also using the cumulative population data; 0 indicates no relative inequality and values closer to +100 favour the rich while values closer to −100 favour the poor

All interventions (with the exception of ORT) exhibited pro-rich inequality patterns where coverage was more concentrated among the rich in the population. Details on the absolute and relative inequalities by intervention are highlighted below.

Absolute gaps between the richest and poorest quintiles were widest for ANCS (52 %, 57 %) and SBA (61 %, 65 %) using both the Q5-Q1 and the SII summary measures, respectively (Table 2). Together with ANC4, SBA and ANCS were also the least equitable of all interventions using relative summary indices. According to the Q5/Q1 ratio, coverage was 5.6 times, 4.9 times, and 3.0 times higher in the richest relative to the poorest quintiles, respectively. Corroborating these findings, the CIX values of 36 %, 31 % and 21 %, respectively, suggested higher coverage among the richest. ANCS was one of the most inequitable interventions despite moderate overall coverage levels. ANC4 had the lowest coverage of all intervention indicators and moderate to high levels of absolute and relative inequality. SBA ranked fourth lowest in overall coverage, highest in absolute inequality, and second highest in relative inequality.

The most equitable interventions across all four equality indices were EIBF and ORT. National levels of early breastfeeding were second highest relative to other interventions (54 %), while the indicator ranked lowest in both Q5-Q1 and SII absolute inequality indices (2 %, 0.4 % respectively), and second lowest in the relative Q5:Q1 ratio (1.04) and CIX (0.5 %). Oral rehydration therapy reached almost 46 % national coverage levels, and was the only indicator which exhibited pro-poor patterns. The absolute coverage difference, though low (−6 %), indicated higher coverage in the poorest quintile (47 %) relative to the richest (41 %), and the SII followed suit. Similarly, though relative inequality was low, the Q5:Q1 ratio was 0.87 and CIX was 0.2 % (confidence intervals covering negative values: −3.3 %, 3.6 %) reflecting, again, slightly higher coverage among the poor. These two indicators exemplified the best combination of moderate/high coverage and low inequalities among all 11 interventions.

Despite having the highest national coverage of evaluated indicators, CPNM (61 %) had moderate levels of absolute inequality (19 %, 20 %, for Q5-Q1 and SII respectively) and the third lowest levels of relative inequality (1.42, 5.4 %, for Q5/Q1 and CIX respectively). Similarly, VITA had moderate average interventions coverage (51 %), and almost identical inequality patterns.

DPT3 and MSL vaccination exhibited low to moderate levels of national coverage, and modest ranks of absolute and relative inequalities. Full vaccination among children under 5 exhibited minimal levels of absolute and relative inequality across all indices and also had very low national coverage (18 %), thus suggesting that children in all wealth groups suffer equally from incomplete immunisation.

### Patterns of inequality

**Fig. 3:**
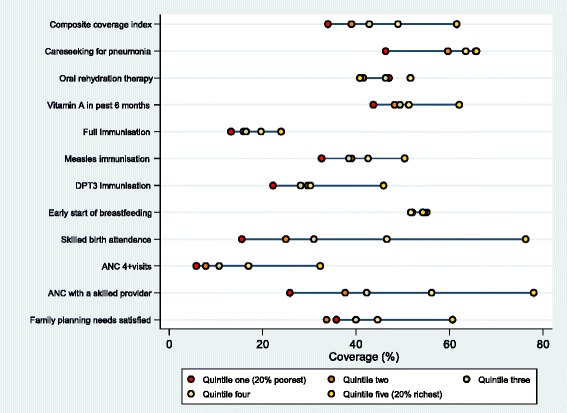
Five‐dot chart showing the interventions & composite coverage index by wealth quintile

Studying the distance in coverage between wealth groups can help uncover patterns such as “linear”, “bottom”, and “top” inequality which can be used for more efficient targeting and programming to reduce inequalities [19, 23]. Figure 3 five-dot plot displays estimates of interventions coverage and the CCI by wealth quintiles. A linear pattern exists when the distance between all wealth groups is similar, while bottom and top inequality are present when gaps are widest among the lower and higher wealth quintiles, respectively [19, 23]. FPS, ANCS, ANC4 and SBA all displayed wide gaps between wealth groups and top inequality patterns. Considering that mean coverage is suboptimal and inequitable even among the rich, mass population dissemination strategies should be considered for scaling up these interventions across the country [19]. For SBA and ANCS, coverage for the richest quintile reached almost 80 % coverage, and thus efforts should be channelled primarily to other quintiles. Minimal variation existed between wealth groups for EIBF, however national coverage was suboptimal. Hence, efforts for encouraging early breastfeeding should be widespread and span all population subgroups. DPT3, MSL, FULL, VITA and ORT also demonstrated slight top inequality and were coupled with inadequate overall coverage. Such patterns are again suggestive of population-wide strategies for scaling up of these interventions. Considering some evidence of linear inequality for MSL and FULL, additional efforts should be made to specifically target the poor to avoid a bottom inequality pattern from evolving [19]. CPNM is the only indicator that displayed bottom inequality patterns suggesting that most of the population is already on its way to reaching optimal coverage while the poor lag behind – such trends underscore the need to specifically target the poorest quintile for this intervention [19].

### Composite coverage

**Fig. 4:**
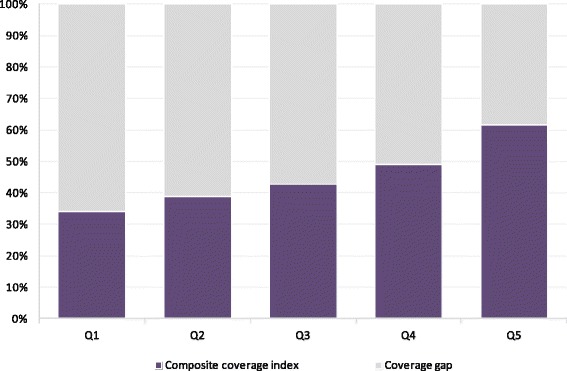
Composite coverage of selected interventions and corresponding coverage gap (needed to reach universal coverage) by wealth quintile

The composite coverage across 8 essential interventions in Afghanistan was roughly 45 % indicating that only about half of the population is receiving adequate coverage. As the CCI is a weighted average of several interventions, inequalities fell within the range of its constituent indicators presented in Table 2. Specifically, the CCI displayed moderate absolute inequality and low levels of relative inequality (Table 2, Fig. 2). The composite indicator displayed top inequality patterns across wealth quintiles indicating that richer individuals were slightly better off in coverage, while others lagged behind (Fig. 3). The corresponding CCI coverage gaps across wealth quintiles were approximately 66 %, 62 %, 57 %, 53 % and 38 %, for Q1 to Q5 respectively, underscoring that gaps to reach 100 % universal coverage were substantial among all socioeconomic subgroups (Fig. 4).

### Co-coverage

**Fig. 5:**
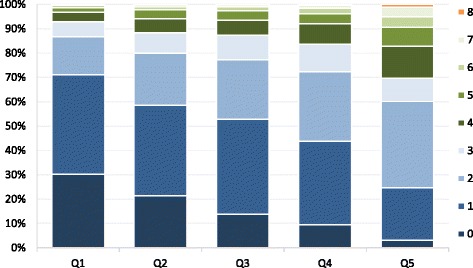
Co‐coverage of health interventions: percentage of children aged 1‐4 years according to the number of key child‐survival interventions received by wealth quintile. Note: Interventions taken into account for the co‐coverage analysis: (1) antenatal care, (2) mother immunised against tetanus, (3) skilled birth attendant, (4) BCG immunisation, (5) 3 doses of DTP, (6) measles immunisation, (7) vitamin A, (8) household with improved drinking water source

Figure 5 displays the proportion of children 1–4 years receiving between 0 to 8 essential child survival interventions by wealth quintile. Substantial inequalities were noted across the quintiles with children from the poorest families receiving the lowest number of interventions. In the lowest quintile, almost 30 % of children received 0 interventions and another 40 % received only 1 intervention; while in the richest quintile, these proportions were about 3 % and 22 %, respectively. The proportion of children receiving 3+ or 6+ interventions was low across all wealth quintiles. About 13 % of the poorest children received 3+ interventions, while only about 2 % received 6 or more lifesaving interventions. Conversely, among the richest subgroup, more children received 3+ or 6+ essential interventions (40 %, 10 %, respectively), however the numbers remained low.

### Regional coverage and inequalities

**Fig. 6:**
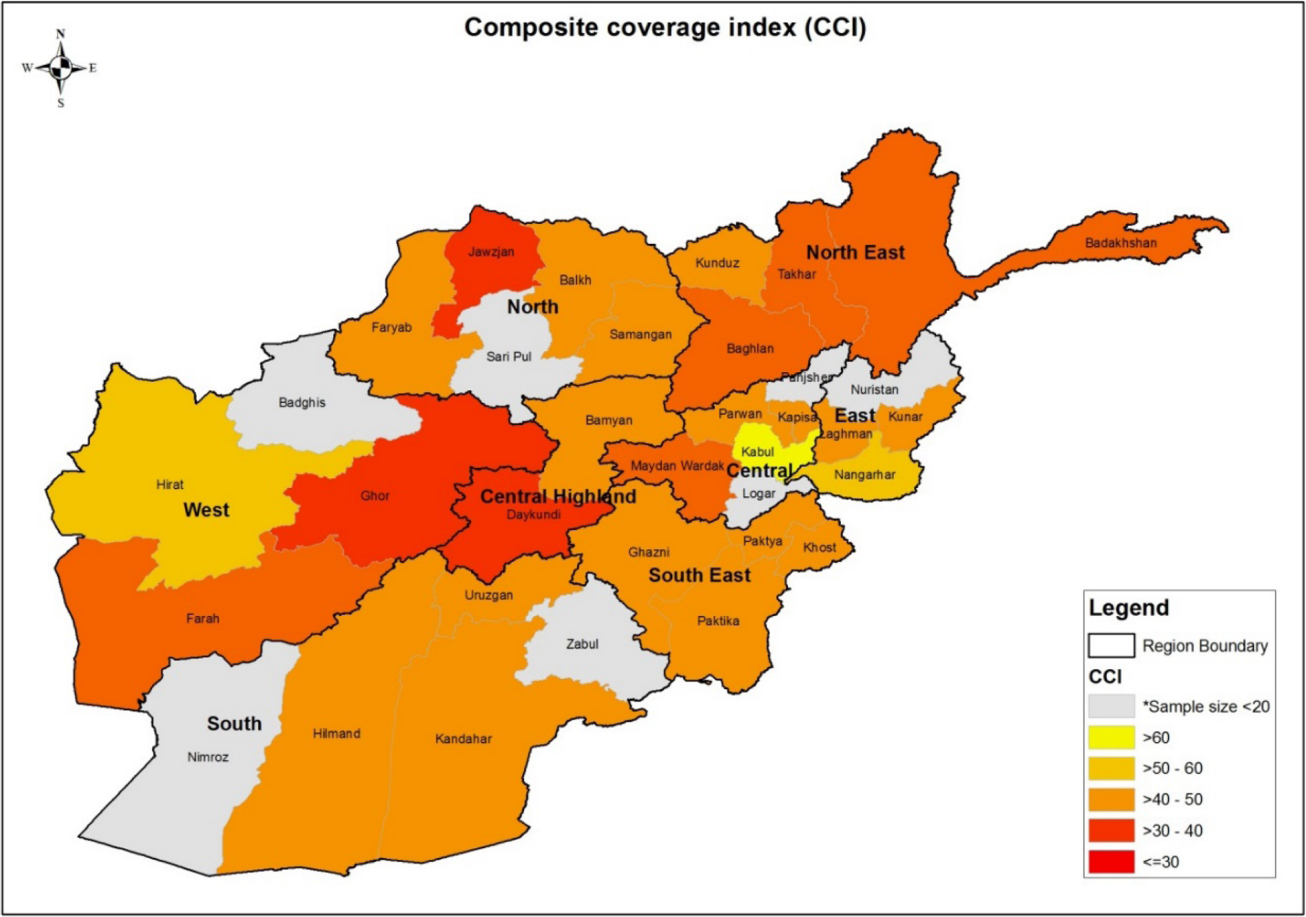
Composite coverage index across provinces and regions in Afghanistan

Disaggregating interventions into subnational populations in Afghanistan revealed important geospatial coverage inequities. Figure 6 displays a clear gradient in the CCI across provinces and regions with available data. Composite coverage was greater than 50 % in regions and provinces with populous urban hubs such as Kabul, Nangarhar and Herat, while some provinces in the less accessible Northern or Central Highland regions had less than 30 % coverage. Several very remote and isolated provinces lacked ample sample size to calculate reliable coverage estimates, however it is likely that interventions reach is even lower in those geographic areas (e.g. Nuristan, Sari Pul).

**Fig. 7:**
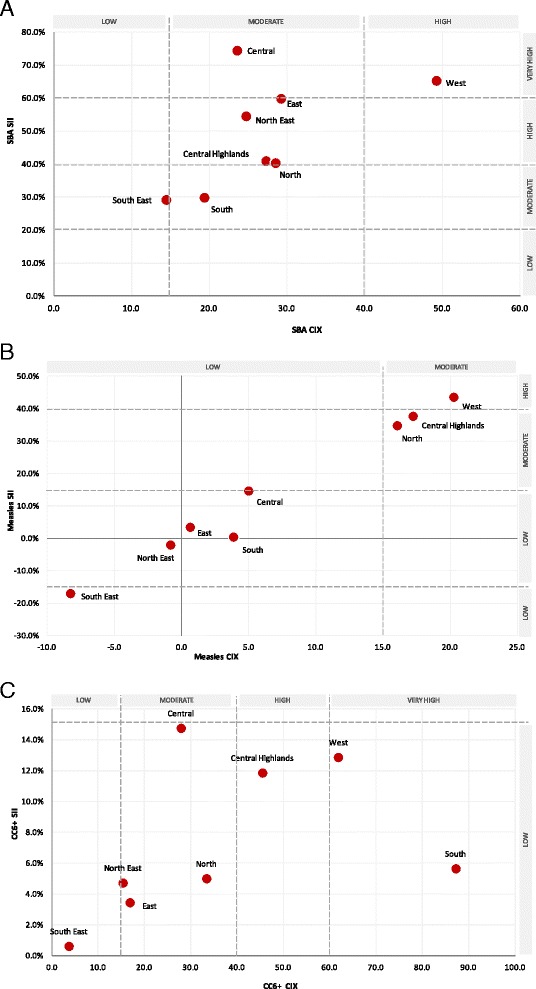
Slope index of inequality (SII) and concentration index (CIX) estimates by region for skilled birth attendance, measles vaccination, and co-coverage 6+. **a**: Skilled birth attendance. **b**: Measles vaccination. **c**: Co-coverage 6+

Table 3 presents average coverage and 95 % confidence intervals by region for SBA, measles, and co-coverage (6+) indicators. Figure 7a-c visualises SII against CIX for each indicator and thresholds are delineated into low (values < |15|), moderate (values |15–40|), high (values |40–60|), and very high (values > |60|) quadrants.

**Table 3:** Coverage of select interventions in eight regions of Afghanistan

	SBA (%)			Measles (%)			Co-Coverage 6+ (%)		
Region	%	LCL	UCL	%	LCL	UCL	%	LCL	UCL
Central	60.8	53.1	68.5	50.8	44.1	57.4	9.1	7.5	10.8
Central Highlands	25.4	18.4	32.4	38.3	28.9	47.7	3.6	1.6	5.6
East	34.7	24.6	44.8	48.9	41.4	56.4	3.1	1.8	4.4
North	23.2	17.6	28.8	35.8	28.5	43.0	2.4	1.7	3.2
North East	37.7	31.8	43.7	46.7	36.0	57.4	4.8	3.3	6.2
South	20.5	11.9	29.2	18.4	7.9	29.0	0.4	0.1	0.7
South East	34.2	20.8	47.7	38.5	24.7	52.3	2.5	1.1	3.8
West	22.2	14.1	30.3	37.0	26.1	47.9	2.7	1.3	4.0

*Estimates presented as means with 95 confidence intervals

The availability of a skilled attendant at birth was low across most regions, ranging from 22 % (West) to 35 % (East). The exception to this was the Central Zone which at about 60 % coverage had higher SBA reach relative to other regions (Table 3). Inequality patterns for SBA were pro-rich (i.e. higher coverage among the rich compared to poor) for all regions and across both absolute and relative measures. The Central region ranked very high for absolute inequality in SBA, along with the East and West regions (Fig. 7a). The North East, Central Highlands and North had high levels, while the South and South East exhibited moderate absolute inequality. Relative inequality tended to be lower than absolute inequality on average for this indicator with most regions exhibiting moderate CIX levels, and only the West ranking high (CIX = ~50). Overall, the South East and South regions appeared to be the most equitable in SBA (ranking low on both CIX and SII), and the West was the least equitable (Fig. 7a).

Measles coverage was lowest in the Southern region of Afghanistan (18 %). Though coverage rates were higher in the remaining regions (range 36 % in the East to 51 % in Central) reach remained substandard and below universal coverage (Table 3). Several regions exhibited low levels of pro-rich relative and absolute inequalities in MSL vaccination including the Central, Southern and Eastern populations (Fig. 7b). The North East and South East also displayed low levels of both inequalities, though trends were slightly pro-poor. The North and Central Highlands had moderate levels of pro-rich relative inequality and higher but also moderate levels of absolute inequality. Together with the West, these three regions were the most inequitable regions in the country for MSL intervention.

Co-coverage of 6+ interventions was extremely low across all regions in Afghanistan, ranging from 0.4 % in the South to 9 % in Central (Table 3). Most regions had co-coverage between 2–4 %. Inequalities in this indicator were pro-rich for all regions (Fig. 7c). The slope index of inequality was less than 15 % for all regions, thus indicating low levels of absolute inequality across the country. In terms of absolute differences, the least inequitable regions were the South East (SII = 0.5 %), East (SII = 4 %), North East (SII = 5 %), North (SII = 5 %), and the South (SII = 6 %). The Central region ranked highest in SII (almost 15 %) followed by the West (13 %) and Central Highlands (12 %). Relative inequality was more pervasive than absolute inequality, with three regions noted as ranking high or very high in the CIX: South (CIX = 87), West (CIX = 62) and Central Highlands (CIX = 45). Most other regions had modest relative inequality levels, while, with a CIX value of 4, the South East ranked lowest.

### Impact of intervention scale-up in inequities in Afghanistan

**Fig. 8:**
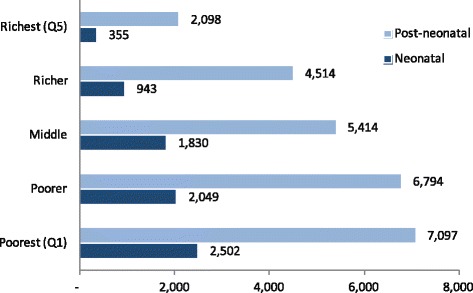
Child deaths prevented by wealth quintiles through scaling up of community level interventions

Our LiST analysis revealed that impact of all community based interventions is greatest in the poorest quintiles with the proportion of lives saved declining as wealth status improved (Fig. 8, Table 4). About 7700 newborns could be saved by scaling up newborn interventions to 90 % by 2025- almost 20 % mortality reduction from 2016. Approximately 60 % of neonates saved would be in the poorest two quintiles, with another quarter in the middle quintile. The most effective interventions include newborn thermal care and oral antibiotics for neonatal infection which together avert almost 60 % of all newborn deaths in the first three quintiles; additionally, application of chlorhexidine could save another 20 % of newborns in Q1-Q3 (Additional file 1-B1). In the richest two quintiles, chlorohexidine and breastfeeding promotion save the largest of number of newborns with about 50 % of neonatal survival gains (Additional file 1-B1). Under the same scale-up scenario, nearly 26,000 post-neonates could be saved by 2025- an approximate 42 % reduction in deaths from baseline (Table 4). Substantial gains can be made in almost all wealth groups (17–27 % reduction across Q4-Q1), though lives saved are greater amongst the poorer. The most impactful interventions for saving post-neonatal lives in Afghanistan are similar across all wealth quintiles (Additional file 1-B2). Handwashing with soap and care of sick children (treatment with ORS, antibiotics for pneumonia, therapeutic feeding for severe wasting) save about 80 % of post-neonatal lives within each wealth quintile.

**Table 4:** Child deaths prevented by wealth quintiles through scaling up of community level interventions

Wealth Quintile	Neonatal				Post-neonatal			
	Baseline	Deaths Prevented	% of deaths averted from base year death	% of deaths averted from total live saved	Baseline	Deaths Prevented	% of deaths averted from base year death	% of deaths averted from total live saved
Poorest (Q1)	10,895	2,502	23	33	13,468	7,097	53	27
Poorer	9,506	2,049	22	27	13,559	6,794	50	26
Middle	9,671	1,830	19	24	12,566	5,414	43	21
Richer	8,074	943	12	12	11,967	4,514	38	17
Richest (Q5)	5,022	355	7	5	10,615	2,098	20	8
*Total*	*43,168*	*7,679*	*18*	*100*	*62,175*	*25,917*	*42*	*100*

## Discussion

To our knowledge, this is the first effort to systematically and comprehensively evaluate socioeconomic inequalities in RMNCH interventions nationally and by geographic regions in Afghanistan. Our results underscore evidence of significant social, economic, and geographic health service inequities among women and children in the country.

We found that national coverage was suboptimal for all interventions studied (around 60 % or less), and particularly low for those requiring multiple interactions with a health care provider or facility such as 4 or more ANC visits (15 %) and full immunisation among children (18 %). These findings are not surprising and are in line with previous assessments of health service utilisation in Afghanistan [7, 8], and analyses from multiple low and middle income countries [24] which have found low access to and use of healthcare services in extreme poverty-stricken, fragile contexts. Nevertheless, from a completely shattered health system in 2001 and against a perpetual backdrop of unstable governance, conflict and violence, environmental disasters, geographical constraints, and extreme poverty- Afghanistan has performed quite well in rapidly scaling up coverage of essential interventions to its civilians. Championed by the MoPH and implemented primarily by contracted non-governmental organisation (NGO) partners, the introduction of the Basic Package of Health Services (BPHS) in 2002 and the Essential Package of Hospital Services (EPHS) in 2005 have been the cornerstones to the progress made in Afghanistan [5]. Efforts should now focus on ways to enhance service coverage and utilisation, health system performance and quality, and cost-efficiency of service provision to the Afghan population. In this regard, a focus on reducing inequities may yield best returns.

Our analysis of the composite coverage index revealed that though only half of the population is benefiting from eight essential preventive and curative interventions, coverage is even lower among less wealthy households. From a total of nine community and facility-based preventative indicators, the proportion of children receiving less than two increased monotonically from 25 % to 70 % from the richest to the poorest quintile- that such a large percentage of children are receiving at most only one life-saving intervention is alarming. Even more disconcerting is that about 30 % of children in the poorest quintile receive no preventative interventions. These results speak to the general lack of adequate care for children and women in Afghan communities, particularly among the poorest.

Not surprisingly, our results underscore significant pro-rich socioeconomic absolute and relative inequalities in all interventions, except curative indicators such as ORT which were slightly pro-poor. This is contrary to an analysis of nationally representative data from 2005 which found no differences in access to public healthcare facilities between the rich and poor [8]. However Trani and colleagues acknowledge that the now outdated 2005 dataset was from a time where Afghanistan’s health system rebuilding was just ramping up and service delivery was low throughout the uniformly poor country, thus predating the evolution of services and consequent socioeconomic disparities in coverage. Results from another 2004/05 survey based on households within BPHS health facility catchment areas found that rates of care-seeking behaviour for illness was high (about 90 %) across all wealth quintiles, and health service utilisation was slightly pro-poor [7]. However this survey was not representative of the general Afghan population and excluded remote and inaccessible districts which are often the most poor. Moreover, because respondents were surveyed within the accessible surroundings of public health facilities, rates of utilisation were likely higher and more similar across populations [7]. Slight pro-poor patterns may have emerged due to the rich often seeking care in other district hospitals or private facilities which are perceived to have better quality of care [7]. Using data from a robust nationally and regionally representative survey conducted in 2010/11, our results highlight that significant socioeconomic inequalities have begun to take shape in Afghanistan only 5 years later. This falls well in line with the “diffusion of innovations” theory [32] which states that in the absence of targeting, novel interventions are first espoused by the wealthier and later dispersed to the less wealthy counterparts. Though Afghanistan has made concerted effort to scale-up provision of health services to the poor and less accessible populations via NGOs and CHW in remote areas, our results underscore that inequalities remain and in some cases are quite pervasive. In lieu of prioritising strategies to reduce gaps and making intensive effort to reach the most vulnerable populations, these disparities will continue to widen and perpetuate a vicious cascade of inequities within Afghanistan.

The most inequitable interventions in Afghanistan by far are those requiring a functional health system and repeat interactions with skilled professionals. More specifically, coverage of ANCS, SBA, and ANC4 are starkly inequitable where the richest have 3.0–5.6 times higher coverage relative to the poor, and gaps of 32 % - 65 % between the extreme quintiles need to be closed to achieve equality. Of interest is the parallels in inequality of these indicators despite their varying levels of overall coverage; ANCS and SBA having moderate levels of coverage and ANC4 has the lowest coverage of all interventions. This suggests that inequalities occur at all levels of health intervention uptake in Afghanistan societies, and that even the least accessed interventions will reach the wealthy first. Amongst the most equitable and best accessed interventions in Afghanistan is early initiation of breastfeeding. This may have to do with the cultural perceptions of acceptability and benefit to the child, and limited cost of this intervention in a relatively poor nation. Our findings are consistent with recent multi-country assessments of LMICs globally which likewise found that SBA and ANC4 were the most inequitable [1, 22, 24], while breastfeeding interventions [1, 22, 24, 33] were the most equitable interventions across high burden countries.

Treatment of sick children has been found as one of the most equitably distributed interventions across high burden countries [22, 24] and this finding holds true for Afghanistan. The administration of oral rehydration therapy to children with diarrhea is relatively high (46 %) when compared to other indicators and in fact exhibits slight pro-poor patterns. This is likely due to the widespread availability of such therapies in Afghan communities, its ease of use and the minimal monetary cost to families [24]. However, further research is needed to systematically and thoroughly explore the main drivers of RMNCH interventions equalities and inequalities in Afghanistan.

Individual childhood vaccines are relatively well accessed and have low levels of inequality in Afghanistan, yet few under 5 children in the country (18 %) are covered with the complete package of essential preventative vaccinations. Negligible inequalities in this indicator suggest that children across all socioeconomic groups in the country remain at risk of developing common yet preventable diseases. The expanded programme of immunisations in Afghanistan has wide reach through various community-based and door-to-door strategies to disseminate one or multi-dose vaccines, however additional efforts should now be considered to ensure continuity and completion of the indispensable package of childhood vaccines. Packaging vaccines with other RMNCH interventions campaigns may be one solution and has shown to be effective in other low-resource and topographically complicated settings [34].

Our assessment of inequality patterns revealed that most RMNCH interventions in Afghanistan exhibit top inequality patterns resulting in mass population deprivation. These trends speak to the importance of nation-wide strategies targeting all socioeconomic groups for effective scale-up and reach across the country. A recent systematic review [34] summarised interventions that are effective in reducing inequalities in maternal and child health in low and middle income settings, and highlighted effective interventions which could be considered for Afghanistan. Outside of initiatives currently underway to address inequality in Afghanistan [11, 35, 36], a focus on increasing demand through educational campaigns and community-based participatory interventions, particularly for women and head of the households, could be considered for increasing awareness and interest in preventative and curative care-seeking behaviour. Additionally, combined health care interventions delivery programmes could be explored and conditional cash transfer schemes could be revisited and strengthened [34].

Our analysis noted significant variation in coverage and inequalities across various regions of Afghanistan. Composite coverage was lowest in the most remote and isolated regions of Afghanistan such as Northern and Central Highlands, while highest in regions and provinces with major urban hubs such as Nangarhar in the East, Herat in the West, and Kabul in the Central region. These results are not surprising and speak largely to better availability and accessibility of services in urbanised areas. Similarly, though SBA coverage was low across the nation, absolute inequalities were most pronounced in this indicator and were particularly highest in the same densely-populated East, West, and Central zones of the country (>60 % inequality between the richest and poorest). Despite adequate physical accessibility of health services in these regions, such inequities often arise due to numerous factors including financial barriers such as high costs. Though user fee bans were implemented in all BPHS facilities in 2008 [7], out-of-pocket payment for health service provision and commodities remains high and often results in catastrophic expenditure [8]. This presents a particular barrier for the lower income and other marginalised vulnerable populations, such as the traveling nomads, destitute and child workers, and those living in urban slums. Efforts to promote inclusive programmes and policies should be prioritised to target these groups and preclude socioeconomic gaps from widening.

Inequalities in measles vaccination were highest in the West, North and Central Highlands zones while relatively low in others. Though these trends could be attributed to reasons previously discussed, an in-depth assessment of the varying factors at play by subnational region is warranted. The Southeast and South generally had lower inequalities in SBA and measles vaccination relative to other regions. This could have been for a myriad of reasons including the uniformly low coverage of interventions in these regions across all socioeconomic populations. Many assessments have also shown that the level of insecurity has an adverse impact on health service utilisation for the poorest and most vulnerable women and children in Afghanistan [8, 37–39]. Due to the recent resurgence in conflict in many parts of the country, particularly the South and Southeast, many health facilities cannot be accessed and/or are poorly equipped and staffed, or have closed entirely [39]. Community-based interventions and outreach programmes are also severely restricted due to travel constraints in insecure areas and lack of staff morale and willingness to work in unsafe districts. As a result, inequities may be minimal due to general low coverage across the regions. To protect and support the most vulnerable in these volatile zones, the MoPH and partners should heighten investments and programmes in these largely insecure regions to specifically target interventions delivery to the poor and otherwise defenceless populations. Other social and demographic factors that could be important to observed regional differentials are discussed in Table 5. A through assessment of factors leading to coverage differentials and inequalities across regions in Afghanistan is a critical next step to further understanding the challenges facing regions and their populations.

**Table 5:** Geographic, demographic and social factors that impact health service inequity in Afghanistan

Regions in Afghanistan vary dramatically in culture, climate, geography and terrain and socioeconomic conditions- all factors which could impact the levels of health service coverage and inequalities [21]. Provinces in the Southern region suffer from lower literacy and income relative to the rest of the nation, and this is compounded by a deteriorating and unstable security situation that exists not only in the South, but also South East and Eastern regions. Moreover, despite the fact 72 % of total population of Afghanistan is living in rural areas, rural conditions vary across regions and within regions. Geographical barriers such as mountainous terrain and desert pose a threat to equitable delivery and access of health services in many regions, particularly the North and Central Highlands. These factors further impact climate, with some regions experiencing harsh and prolonged winters which adversely impact agriculture and mobility. Scattered populations across the mountains and deserts of Afghanistan are often difficult to reach and as a result exhibit lower coverage of services. Evidently, Afghanistan’s unique and complex geography epitomises conditions which severely convolute and constrain gains in health services access and delivery. Cultural factors vary across regions in Afghanistan; Pashtuns occupy the South, Southeast and Eastern parts of the country, while Tajik, Uzbeks, and Hazara (among others) reside throughout other regions. Varying languages and cultural practices across these unique ethnicities could be factor leading to differences in women seeking and receiving health services for themselves and their children.	

### Implications of findings

Our results present several considerations for health policy and programming in Afghanistan. In addition to recommendations stated above, movements for improving health worker availability in remote areas and to marginalised populations across regions in Afghanistan is critical. The existing community health worker deployment programmes have shown promising utility in improving health care access and outcomes among the poor in Afghanistan [5], and could be expanded and repurposed with a specific focus on reaching the unreached and reducing inequalities. A recent cross-sectional evaluation by Alonge and colleagues [17] found that areas with higher numbers of CHW had greater gains in reducing health service inequalities between the rich and poor in Afghanistan. However a thorough assessment by region was not conducted and would be a valuable next step. Modeled strategies have consistently shown higher impacts of community-focused and primary care initiatives in reducing deaths among the poorest sectors of the populations, hence reducing disparities [40, 41]. Safety nets and mobile clinics/outreach teams have been shown as effective in reaching marginalised populations in Afghanistan [5] and should also be further emphasised for hard-to-reach populations in Afghanistan. We reiterate the need for special considerations for conflict and insecure areas, without which, health service access and utilisation will remain hopelessly low in these regions. Negotiations for health service access in conflict-prone districts should be stressed by the Afghan government, funders and implementation partners, especially considering the large number of civilians impacted by diffuse conflict spread and frequency in the nation today. In addition to priorities discussed above, efforts focused on targeting at-risk groups such as young girls, especially those married and pregnant, must not be lost. About 46 % of marriages in Afghanistan occur amongst young children under the age of 18 years- some of the highest rates of child marriage worldwide [42]. Considering the heightened risks of exploitation, domestic abuse, and adverse pregnancy-related outcomes among adolescent girls [42], specialised efforts to reach and effectively intervene for youth females must be at the forefront of agenda-setting in Afghanistan. Many health problems and behaviours such tobacco and drug use and poor diet arise during adolescence for boys and girls. These have serious impact not only on their current health and well-being but also longer term into adulthood; considering Afghanistan’s overwhelmingly young population (>50 % of the population is less than 15 years of age), this group must be targeted in national programmes and policies to promote and safeguard their future as productive members of civilian population in Afghanistan. The 48–72 h period between labour and delivery is especially critical for maternal and newborn survival and preventing still births, and particularly so for young mothers; thus interventions in these critical time window should be emphasised for effective scale-up nation-wide. The Every Newborn Action Plan (ENAP) [43] and interventions to reduce stillbirths [44] provides an evidence-based road map that could be consulted and adapted to reduce perinatal mortality in Afghanistan. Along the same lines, the Global Action Plan for Pneumonia and Diarrhea (GAPPD) [41] should be incorporated into initiatives for improving child survival, particularly In light of the tremendous number of preventable child deaths from infectious pneumonia and diarrhea in the country [5]. Our analysis also supports key strategies to reduce the negative impact of poor social determinants of health in Afghanistan such as poverty, food security and undernutrition. Potential safety nets such as poverty alleviation programmes and cash transfers are another option that may be considered in addition to food security programmes.

### Limitations

Strengths of this analysis have been previously mentioned, they include the thoroughness in terms of interventions assessed, the completeness of socioeconomic inequality types and indices explored, and the large and representative sample sizes of the MICS 2010/11 survey which permitted estimates of the country as whole and regionally. However, several limitations must be noted. First, selection bias resulting from responders who agree to participate in the survey could be present; however because the MICS 2010/11 response rate was about 99 %, the impact of such biases should be nil. Indicators used in this study were primarily from maternal survey reporting which could be subject to recall bias. Biases could be differential and/or non-differential and further work is being done to understand these biases in standardised health surveys. The use of asset indices does present some limitations, for instance, the equivocal selection of assets for constructing the index, strong correlations between wealth and rural vs urban disparities, and other considerations which have been discussed elsewhere [22]. Despite these limitations, asset indices remain the preferred method of exploring gaps between the rich and poor in LMICs [22].

### Future research

A thorough analysis of the facilitators and barriers of RMNCH intervention access and the determinants of inequities in the unique regions and provinces of Afghanistan is needed to guide effective strategising and programming at the province and district level. Further disentangling the barriers amongst the poorest, whether financial, social, cultural or geographical, would be a natural next step towards understanding and targeting the challenges to achieve universal coverage. Concerted efforts should also be made to design and test effective interventions that increase overall coverage such as through universal health care, while reducing inequalities in Afghanistan’s unique context. Combination of approaches to reduce poverty barriers such as cash transfers, voucher schemes and community empowerment strategies should also be explored. Future research should also consider exploring socioeconomic inequalities in mortality, morbidities and nutrition outcomes to further understand how differentials in essential interventions coverage translate into health and survival inequities in Afghanistan. Additionally, examining other dimensions which lead to health inequities, including ethnicity, gender, education and urbanisation, would also prove valuable for understanding the totality of disparities affecting Afghan society. Better data is also critical to planning and could guide interventions and strategies such as community health worker programmes. Our analysis was not able to explore inequalities at the provincial or district level in Afghanistan due to limited data for robust equity analysis at this granularity. The decentralised MoPH has provincial directorates which form and execute local policies and programmatic efforts which could benefit from province level information for better targeting. Composite coverage indicators were also difficult to assess for some sparely populated and/or inaccessible provinces [21] such as Badghis, Sari Pul, Nimroz, Zabul, Logar, Panjsher and Nuristan which provided minimal data for province-level estimates. Better and more granular data should be collected as it is important for province and district level estimates. The forthcoming Afghanistan demographic and health survey may also provide granular information that can assist in tracking progress.

## Conclusions

Afghanistan has been making much progress in extending reach of RMNCH services in the country, however inequities remain pervasive and pose a threat to additional gains. In a country where pronounced and exacerbated inequities prevail across social and economic population subgroups and subnational geographies, targeted efforts to reach the disadvantaged will prove most valuable for accelerating health gains. This goal has been recognised by the MoPH and country partners, who have included equity in the broader mission statement to “improve the health and nutritional status of the people of Afghanistan in an equitable and sustainable manner through quality health services provision, advocating for the development of healthy environments and living conditions; and the promotion of healthy lifestyles.” Despite competing priorities, moving forward, Afghanistan will need continuous support from global organisations, donors, government, private sector, and civil society, to focus on reducing inequalities to promote equity and justice for all. In what is foreseen as the most unstable and volatile decade to come in Afghanistan’s recent history, focussing efforts on the most vulnerable populations’ poor will be paramount to sustaining and enhancing gains in the SDG era.

### Key messages

1. Coverage of essential maternal and child health interventions is unacceptably low in Afghanistan, and pro-rich socioeconomic inequalities are widespread
2. Facility-based interventions including antenatal care and skilled birth attendance are the most inequitably distributed, while child vaccinations and breastfeeding interventions are least inequitable
3. Wealth disparities, geographical remoteness/physical barriers, and insecurity pose a threat to achieving universal coverage of health interventions in Afghanistan especially in the South, South East, North and Central Highlands; these barriers should be further understood and targeted for effective interventions scale-up.
4. Reducing inequities should be central to national and international programming and policy initiatives to improve health status and survival of Afghans and would require innovative and evidence-based strategies to reduce disparities.
5. Improving health worker availability, introducing safety nets, and increasing deployment of mobile clinics/outreach teams to remote and hard to access areas have shown promising utility in Afghanistan and could be further scaled up to reduce inequities.

## Abbreviations

ANC4, Antenatal care visits (4 or more); ANCS, Antenatal care with a skilled provider; BCG, Baccille Calmette Guérin vaccine; BPHS, Basic package of health services; CCI, Composite coverage index; CHW, Community health worker; CIX, Concentration index; Countdown, Countdown to 2015; CPNM, Care seeking for children with suspected pneumonia; DPT3, Diphtheria, pertussis, tetanus vaccine (3 doses); EIBF, Early initiation of breastfeeding; ENAP, Every newborn action plan; EPHS, Essential package of hospital services; FPS, Family planning needs satisfied; FULL, Full immunisation; GAPPD, Global action plan for pneumonia and diarrhea; HDI, Human development index; LiST, Lives saved tool; LMIC, Low and middle income country; MDG, Millennium development goals; MICS, Multiple indicator cluster survey; MMR, Maternal mortality rate; MoPH, Ministry of public health; MSL, Measles vaccine; NGO, Non-governmental organisation; NMR, Neonatal mortality rate; ORS, Oral rehydration solution; ORT, Oral rehydration therapy and continued feeding; Q, Quintile; RMNCH, Reproductive, maternal, newborn and child health; SAM, Severe acute malnutrition; SBA, Skilled birth attendant; SDG, Sustainable development goal; SII, Slope index of inequality; TT2, Tetanus toxoid 2+ doses during pregnancy; U5MR, Under-5 mortality rate; VITA, Vitamin A supplementation; WA, Improved drinking water sources

## Additional file

Additional file 1: Additional data, tables and figures. (DOCX 52 kb)
