# Supplementary material for: Coverage and inequalities in maternal and child health interventions in Afghanistan
Source: BMC Public Health. 2016 Sep 12;16(Suppl 2):797. doi: 10.1186/s12889-016-3406-1 (PMC5025831; doi:10.1186/s12889-016-3406-1)
Supplement: Additional file 1: — Additional data, tables and figures. (DOCX 52 kb) [file 12889_2016_3406_MOESM1_ESM.docx]

**Coverage and inequalities in maternal and child health interventions in Afghanistan**

**Additional file 1**

Table of Contents

[A. Definitions of Indicators Used in the Analysis 2](#_Toc446868313)

[B. Lives Saved Tool Results 3](#_Toc446868314)

[B.1 Neonatal deaths averted by intervention 3](#_Toc446868315)

[B.2 Post-neonatal deaths averted by intervention 3](#_Toc446868316)

# Definitions of Indicators Used in the Analysis

| **Indicator** | | **Definition** |
| --- | --- | --- |
| **1.FPS** | *Family planning needs satisfied* | Percentage of currently married fecund women who say that they do not want any more children or that they want to wait 2 or more years before having another child, and are using contraception (met need for contraception divided by the demand) |
| **2.CPR** | *Contraceptive prevalence rate* | Prevalence of current contraceptive use among married women 15-49 years old, any method (%) |
| **3.ANCS** | *Antenatal care visit by skilled provider* | Proportion of mothers who were seen by a skilled health provider in at least one antenatal care visit during last pregnancy |
| **4.ANC4** | *4+ antenatal care visits* | Proportion of mothers who had at least 4 antenatal care visits during last pregnancy |
| **5.SBA** | *Skilled birth attendant* | Proportion of mothers who had their delivery assisted by a skilled health professional |
| **6.TT2** | *Maternal tetanus-toxoid vaccine 2+ doses* | Proportion of mothers who received at least 2 doses of tetanus-toxoid vaccine during last pregnancy |
| **7.EIBF** | *Early initiation of breastfeeding* | Proportion of newborns put to the breast in their first hour of life. |
| **8.ITNC** | *Insecticide‐treated bed net for children* | Proportion of children aged 0–59 months who slept under an ITN the night before the interview (Not Available in MICS 2010) |
| **9.DPT3** | *DPT immunization* | Proportion of children aged 12‐23 months who received three doses of diphtheria/pertussis/tetanus vaccine |
| **10.MSL** | *Measles immunization* | Proportion of children aged 12‐23 months who received a dose of measles vaccine |
| **11.BCG** | *BCG immunization* | Proportion of children aged 12‐23 months who received Baccille Calmette Guérin vaccine |
| **12.FULL** | *Fully immunized children* | Proportion of children aged 12‐23 months who received three doses of DPT and Polio vaccines and one dose of BCG and measles vaccines |
| **13.VITA** | *Vitamin A supplementation* | Proportion of children aged 6–59 months who received at least one high dose of vitamin A supplement in the previous six months |
| **14.CPNM** | *Careseeking for pneumonia* | Proportion of children aged 0–59 months with suspected pneumonia taken to an appropriate health provider |
| **15.ORT** | *Oral rehydration therapy* | Percentage of children aged 0–59 months with diarrhea in the previous two weeks who received oral rehydration therapy (packets of oral rehydration salts, recommended home solution, or increased fluids) and continued feeding |
| **16.WA** | *Access to improved water* | Percentage of the households using improved drinking water sources (including piped on premises, public standpipe, borehole, protected dug well, protected spring, rainwater collection) |

# Lives Saved Tool Results

## B.1 Neonatal deaths averted by intervention

| **Intervention** | **Poorest** | **Poorer** | **Middle** | **Richer** | **Richest** |
| --- | --- | --- | --- | --- | --- |
| Micronutrient supplementation (iron and multiple micronutrients) | 277 | 227 | 211 | 160 | 56 |
| Breastfeeding promotion | 322 | 315 | 275 | 226 | 79 |
| Chlorhexidine | 475 | 378 | 345 | 260 | 93 |
| ORS - oral rehydration solution; | 75 | 75 | 68 | 60 | 37 |
| Thermal care | 701 | 541 | 468 | 121 | 53 |
| Oral antibiotics for neonatal infection | 655 | 513 | 466 | 117 | 40 |

## B.2 Post-neonatal deaths averted by intervention

| **Intervention** | **Poorest** | **Poorer** | **Middle** | **Richer** | **Richest** |
| --- | --- | --- | --- | --- | --- |
| Micronutrient supplementation (iron and multiple micronutrients) | 35 | 34 | 25 | 19 | 12 |
| Breastfeeding promotion | 373 | 541 | 404 | 382 | 153 |
| Appropriate complementary feeding | 265 | 230 | 219 | 177 | 101 |
| Vitamin A supplementation | 212 | 175 | 147 | 110 | 40 |
| Hand washing with soap | 1,653 | 1,565 | 1,306 | 1,028 | 478 |
| ORS - oral rehydration solution | 916 | 920 | 763 | 618 | 304 |
| Oral antibiotics for pneumonia | 1,614 | 1,433 | 928 | 882 | 397 |
| Zinc - for treatment of diarrhea | 252 | 231 | 188 | 148 | 61 |
| Therapeutic feeding - for severe wasting | 1,236 | 1,202 | 960 | 759 | 359 |
| Treatment for moderate acute malnutrition | 417 | 380 | 386 | 323 | 139 |
